# Student Misbehaviour and Teacher Coercion. A Comparative Study of Contextual Factors in Primary and Secondary Education Teachers

**DOI:** 10.3390/ijerph17249429

**Published:** 2020-12-16

**Authors:** Santos Orejudo, Juan-José Navarro, Eva Vicente, María Jesús Cardoso

**Affiliations:** 1 Department of Psychology and Sociology, Faculty of Education, University of Zaragoza, 50009 Zaragoza, Spain; sorejudo@unizar.es (S.O.); jjnh@unizar.es (J.-J.N.); 2 Department of Psychology and Sociology, Faculty of Health of Sciences, University of Zaragoza, 50009 Zaragoza, Spain; mcarmor@unizar.es

**Keywords:** student misbehaviour, classroom management, primary school teachers, secondary school teachers, Spain

## Abstract

This study analyses the relation between student misbehaviour and teacher coercion from a teacher perspective by taking further contextual variables into account. Our participants were 480 male/female secondary education and 351 primary education teachers from the Spanish Autonomous Community of Aragón (Spain). This study forms part of the 2017 Coexistence Study in Aragón Education Centres. According to the theoretical framework and the SEM (structural equation modeling), the results revealed a close relationship between student misbehaviour and teacher coercion, although other contextual variables also appeared in the regression equation: in coexistence rules and in teacher competence. We ultimately found a certain degree of difference between the primary and secondary education levels. On the secondary school level, teacher conflicts were associated with student misbehaviour, while coexistence rules and participative and inclusive activities predicted teacher coercion. Conversely, on the primary school level, participative and inclusive activities predict a lower frequency of student misbehaviour, while teacher competence predicts a lower frequency of teacher coercion.

## 1. Introduction

School life involves a high diversity of stakeholders: students, teachers, management teams, parents, support personnel, services, administration, managers of out-of-school activities associated with schools, and others. Research has centred on peer relationships in recent years as a result of the impact caused by cases of bullying. However, it is also necessary to continue to pay attention to asymmetrical student–teacher relationships, because they are just as important in the classroom environment, as well as in terms of coexistence at school. One way of dealing with these relationships is to analyse student–teacher interactions via the construct of so-called disruptive conduct or misbehaviour, which can be displayed by both students and teachers alike [1]. Cross-cultural studies [2] have revealed that misbehaviour is a phenomenon that appears in several cultures. The role of leaders, guides, or mediators, which is attributed to teachers in these interactions [3], reinforces the need to examine these relations in more depth from a teacher perspective, as we do in this study.

Such types of conduct have significant repercussions within the education system. Handling students who cause such problems is one of the most influential factors behind teacher stress as far as teachers’ feelings of competence are concerned, even leading certain teachers to abandon their profession altogether [4,5,6,7,8,9]. These problems likewise affect the classroom environment and, therefore, student learning [10].

Problematic student–teacher interactions have been explained through various approaches. One such approach is based on reciprocity between both parties; that is, when faced with student misbehaviour, the teacher responds with the same type of conduct, which interrupts the interaction due to a threat or by making a student leave the classroom [3,11,12]. Nevertheless, not all teachers handle these situations equally. Lewis et al. [13] ascertained that the degree of concern caused by student misbehaviour and the coping strategies applied to face them mediate the application of coercion, punishment, and aggressive strategies. According to Allday [14], a lack of perceived competence to handle these conducts means that even more over-corrective strategies are applied. Likewise, that which teachers attribute to students’ personal and family characteristics, or even to unknown factors [15,16], contributes, to a great extent, to the appearance of negative interactive loops [17]. Evans et al. [4] point out the degree to which teachers’ mental representation of such conducts affects their moods and, perhaps, also their own behaviour. Schaubman et al. [9] experimentally supported this hypothesis: they verified that punishment measures applied to students can be reduced by means of interventions designed to provide teachers with a greater amount of resources to solve their problems.

Other theoretical approaches can nevertheless help us to grasp these relations, and those approaches do not limit themselves to analysing the behaviour of the two involved parties, students, and teachers. They hypothesize that such behaviour arises within the context of the classroom and the school institution; therefore, such behaviour is affected by a series of more widespread factors, which are of an organisational nature [18,19]. Those factors can be analysed from the vantage point of the elements that constitute the “school climate”, e.g., the reigning atmosphere within the school. According to authors such as Bear, Yang, Mantz, and Harris [20], Fatou and Kubiszewski [21], or Wong and Siu [22], the notion of school climate refers to a series of emotional/motivational aspects that exert an influence on students’ well-being or on their academic performance. School climate includes aspects such as the relation between students and teachers, the perception of norms and privileges, the expectations and interests of the teachers and the administrative team, the teachers’ practices, and the academic support as perceived by the students. The relevance of these dimensions in the educational context becomes manifest when a country’s educational system is subjected to a PISA assessment [23]. Teachers are evidently a key agent in the establishment of relations with students [19,24], in the development of teaching-learning activities, in the evaluation of their achievements, in providing material and emotional support in their school, and in looking after the students’ peer–peer relationships in order to encourage harmonious coexistence in the school. Professors are likewise entrusted with the task of applying the school’s norms, while creating channels and means of participation for the students to participate therein [25].

Our goal in this study is, thus, to analyse which variables associated with teacher practice and school context are significantly related with the perception of student misbehaviour and teacher coercion. Among those variables, this study focuses on classroom practices related to inclusive education, relationships established with students, social support as perceived by peers or managers, and rules of coexistence. As these contextual variables may also exert a differential effect at various educational levels (primary vs. secondary), we have carried out this study with the purpose of analysing the relation amongst these factors from a teacher perspective by comparing their predictive power at two different levels of education.

### 1.1. Theoretical Framework

The term “student misbehaviour” comprises a set of behaviours displayed by students, conducts that go against implicit or explicit classroom rules, as well as against what adults expect of their behaviour. Student misbehaviour, thus, requires teachers to intervene in some way [26]. Beaman et al. [27] review this topic and address it in a broader context, calling it “troublesome classroom behaviour” (p. 45). O’Neill and Stephenson [28] describe three groups for such conduct: “(a) disrupted learning and non-compliance; (b) aggressive, antisocial and destructive behaviours; (c) disorganisation (disruptive behaviours and non-compliance, disorganisation, and aggressive, antisocial and destructive behaviours)” (p. 1135). According to Clunies-Ross et al. [29], talking out of turn and hindering other children are conducts that Australian students most frequently display, followed by distracting behaviours, disrupting, disobedience, idleness/slowness, making unnecessary noise, or aggression. In this category, Alstot and Alstot [30] group certain conducts, such as the form of noncompliance, being off-task, not participating in the lesson, showing aggression towards other students and teachers, talking, not paying attention, interrupting, arguing, verbal abuse, unpunctuality, and abuse of equipment. Other more specific types of student misbehaviour can be identified in other contexts, such as the foreign language classroom [31].

Although many of these conducts are considered minor, this does not mean they are irrelevant, particularly in view of the effects they may have in the classroom and the time that teachers spend in their attempts to control them [14,15,27,29]. Amongst other repercussions, the fact that such problematic behaviour takes place in the classroom affects the learning environment [10] and implies that teachers must devote a great amount of time to interrupt the pedagogical flow, which means less time they can invest in teaching in itself [32]. As previously mentioned, student misbehaviour is also a relevant source of teacher stress, which is particularly mediated by the type of strategies that teachers adopt in order to control such situations [29,33]. Such episodes, therefore, occur much less frequently when teachers feel competent at handling them [32].

It is true that teachers may also display unsuitable behaviour to the same extent as students do [1]. Roache and Lewis [11] use the term “teacher misbehaviour” and identify certain conducts in teachers who apply a coercive style, “fundamentally depending on the use of punishments and aggressive teacher behaviour, such as yelling in anger, using sarcasm to belittle students and imposing group punishments for individual infringements” (133). The effects of teacher misbehaviour are also relevant because they have a negative effect on the classroom environment [20] and prevent teachers from teaching citizen or personal responsibility values [11].

### 1.2. Teachers and Misbehaviour in the Classroom

Causes of student misbehaviour can differ; Alstot and Alstot [30] have reviewed the factors related to them. They not only point out personal characteristics of students (personal background, motivation, and family personal factors), but also the classroom context (direct consequences of misbehaviour, classes not meaning anything, and boredom in class). Many of these factors stress the teacher in his/her important role: he/she is faced with the choice of either allowing those problems to continue, or preventing them. The teacher’s role is, thus, relevant (1) for the type of strategies adopted to handle those conducts in the short term, and for (2) the way it influences his/her capacity to plan classroom activities [12].

Regarding (1), e.g., strategies, an analysis of the immediate consequences of student misbehaviour from a functional perspective stresses the importance of teachers’ actions as a key element that allows the student misbehaviour problem to continue. The strategies they adopt are a determining factor. Proactive strategies are related to the ability to better handle such conducts because they are designed to prevent them, whereas reactive-type strategies are associated with coercive teacher conducts becoming more likely and less successful in the long term [3,17,26,29,30,34,35].

Regarding (2), e.g., the planning of classroom activities, teachers can indeed propose certain specific types of activities, which can serve as an appropriate tool to prevent conduct problems. A structured environment, along with clear rules and activities designed to engage students’ attention, can act preventively. Clunies-Ross et al. [29] establish that strategies such as paying attention to alternative behaviours encourage a positive classroom environment and increase the time spent on performing tasks, which could prevent such problematic behaviour from arising.

The interesting observational study by Ratcliff et al. [3] offers some pieces of evidence. After classifying a set of teachers according to other colleagues’ judgements, such as being strong when applying these practices or needing to improve, these authors verified that less student misbehaviour was displayed in the classrooms of the former than in those of the latter. Moreover, the teachers judged as being strong interacted more with students and devoted a greater amount of consideration to further questions and tasks with which to engage them; moreover, the classroom environments they created were more productive. Conversely, in the classrooms of the teachers perceived as needing improvements, a greater amount of student misbehaviour took place, while attempts to control the situation proved less effective, which only led to a perpetuation of problematic student behaviour, thereby causing teacher frustration.

Mundschenk [35] believes that a teacher who effectively handles student misbehaviour in class has gained students’ respect, communicates with them, and performs activities that engage students to accomplish them. Such measures encourage student engagement in class activities and in the classroom, which thereby allows fewer student/teacher misbehaviours and, thus, causes less stress [33]. In schools where programmes designed to encourage such practices have been set up, it has been shown that they can have effective outcomes [33].

### 1.3. Teachers and Social Support

Apart from the practices or strategies applied by teachers in classrooms, another teacher-related factor that might be associated with less frequency or a greater frequency of problematic behaviour is the peer support perceived by teachers. We should not forget that teachers carry out their work in an immediate social peer context, which is directly related with their performance and well-being. In line with this, certain pieces of evidence reveal that support perceived from peers and administrative teams of education centres is significantly related with the specific practices that teachers adopt in classrooms. Tsouloupas et al. [32] propose peers and administration as key elements in teachers’ acquisition of the competence to deal with student misbehaviour. Clunies-Ross et al. [29] reveal that those teachers who adopt more relative-type strategies in classrooms tend to report less social support from peers, and that both conditions (student misbehaviour and lack of social support) are sources of stress. In a work cited above, Lewis et al. [13] applied an SEM (structural equation modelling) model and reported how a lack of social support resources for Australian secondary education teachers predicted the use of coercive, aggressive, and punishment strategies by teachers.

The collective self-efficacy construct also stresses the importance of the peer context in how teachers act to handle student misbehaviour [36]. This construct is defined as “beliefs reflect teachers’ perceptions of group-level attributes; that is, judgements of the capabilities of the staff or school to which they belong” [37] (p. 342). In empirical terms, Sørlie and Torsheim [36] indicate that collective self-efficacy longitudinally predicts the behaviour problems reported by Norwegian primary education teachers. Indeed, less student misbehaviour takes place in education centres in which increased collective self-efficacy is perceived, and the inverse relation is likewise observed.

### 1.4. Rules and Punishments

A third contextual variable could mediate the student–teacher interaction and impact the control exerted on student misbehaviour. We are referring to the rules and punishment systems set up when rules are not followed. Nonetheless, punishment management does not come across as being equally effective [38], and not all teachers apply punishment in the same way. Trotman et al. [39] examined this idea in depth and pointed out that if the causes of such conduct are not well understood and students are not appropriately managed, control measures based on consequences will be inefficient. Roache and Lewis [11] suggest that those teachers who attempt to encourage their relationship with students tend to include dialogue and discussion, maintain a system of consequences whenever required, and obtain more positive results when managing problematic conducts, unlike those teachers who resort to a more coercive teaching style. Similarly, Ratcliff et al. [3] observe that those teachers perceived as being more effective resort to coercion procedures less and employ explicit verbal rewards more to encourage interest being shown in tasks and to improve their relationship with students.

Alstot and Alstot [30] explain that when disciplinary measures must be taken, they need to be based on a functional analysis of conduct in order to be effective. Nonetheless, as O’Neill and Stephenson suggest [28], it is no easy task for a teacher to develop skills that enable him/her to apply contingencies in a suitable manner. These authors analysed the evolution of primary education teachers in their first teaching year and observed that teachers find it easier to acquire confidence to set up less efficient corrective measures than preventive measures. One relevant factor that falls in line with this is in accordance with the type of attributions that teachers make regarding the causes lying behind such conducts, which are almost always more student-centred than teacher-centred, and which are less focused on classroom practices [15].

From the perspective of the education centres, those centres that deal with problematic behaviour by applying clear management systems tend to have fewer problems, particularly when the classroom environment is positive, performance expectations are high, moral values are present, and students are treated well [10]. When this happens, rule systems become another positively perceived classroom environment element [20]. Similarly, these authors indicate that centres differ from one another as to the way they contemplate the application of rules and, in turn, the creation of different education environments.

### 1.5. Teacher-Student Relationship

Another factor associated with the successful management of problematic behaviour, apart from those previously mentioned, is the teacher’s capacity to establish good relationships with students, including those who display such conducts. This variable is related with a lesser frequency of student misbehaviour in the classroom [11,26,30]. The cultivation of a good relationship with students is associated with the teacher profile characterised by using rewards, dialogue, teacher sensitivity, and regard for adolescent perspective, acknowledging own responsibility, and so on [33,40,41]. In general terms, encouraging participation in classrooms by respecting one another and promoting coexistence are good practices that encourage excellent student–teacher relationships and ensure greater teacher competence [33]. The opposite effect has also been noted; teachers who adopt punishment-type strategies involving coercion and control tend to develop worse relationships with those students who display problematic behaviour, mediated by teachers avoiding these students [3,9].

In these contexts, encouraging mutual respect between students and teachers is a key element for maintaining a good student–teacher relationship in classrooms, and can be implemented via specific programmes [33]; it also appears as a product of teachers’ proactive interventions, such as hinting at and taking care of or dealing with students’ needs [26].

As previously mentioned, good relationships with students do not only depend on contextual factors; certain personal factors can also be trained. Regarding this matter, Nizielski et al. [42] indicate that teachers’ emotional intelligence is a mediator of classroom practices in relation with the sound handling of student misconduct. These authors even contemplate that the competence of emotional intelligence may be more relevant for dealing with behaviour problems than with academic, performance-related tasks. The capacity to attend to the needs of students with behaviour problems and to empathise with them would serve as a means to establish such a relationship. In any case, establishing positive relationships with students requires time and availability for interaction, as well as developing the aforementioned skills [9].

### 1.6. Rationale for This Study

Student misbehaviour and teacher coercion are interrelated: together they form an interactive pattern that perpetuates the situation [3,10,11,12,29,33]. Alternative strategies can be a good tool for teachers to avoid the stress generated by such situations [13,14]; however, other factors related with classroom climate could likewise explain those conducts and could help prevent such problems. Previous studies have evidenced the role such factors can play, either in relation with student misbehaviour [3,20,26,29,30,33,35], or in relation with teacher coercion [13,32,36,42]. However, no previous study has analysed their influence on the two variables in conjunction.

In the same way, very few studies have analysed the roles that the level of education plays [15] because most research has centred on the frequency of student misbehaviour, the type of student conducts, or the kind of strategies that teachers adopt [11,27]. However, certain authors [29] indicate that the level of education taught by teachers might be a variable worth bearing in mind when attempting to understand relationships between the role of teachers and their peers and student misbehaviour. What is more, there are very few studies on the subject of student misbehaviour or teacher coercion in Spain. Those that exist tend to focus on the relations of those conducts with other dimensions that affect coexistence, such as bullying [43], teacher expectations, or the relation with family [18]. Those studies generally adopt the vantage point of the student and are mainly focused on the secondary education level.

### 1.7. Objectives and Hypotheses

Bearing this research context in mind, and considering the priority given to studies in the Anglo-Saxon world with fewer articles from other contexts [27], the present study attempts to verify if previously reviewed variables are just as relevant in other cultural contexts, in this case Spain. To do so, the present work analyses the relations between student misbehaviour and teacher misbehaviour, as well as how other teacher-related factors may influence them, namely teachers’ capacity to establish good relationships with students, teachers’ capacity to establish motivational and inclusive teaching strategies, their use of participative and inclusive teaching resources in classrooms, their perceptions of rules and discipline, and teachers’ perceived peer social support context. This study also analyses whether the relation and weight amongst these variables can differ between primary and secondary education teachers, given the two teaching levels’ distinct training profiles as well as the given students’ curriculum and age, thereby giving rise to school climates that can differ considerably among themselves.

Specifically, a first hypothesis in our study envisages that the student misbehaviour measure will be the main predictor of teacher coercion (H1). A second hypothesis proposes that teachers who maintain better relationships with their students tend to establish motivational and inclusive teaching strategies, use a greater amount of teaching resources to encourage student participation, have a sound perception of coexistence rules, and receive a greater amount of social support from their peers. They will, thus, observe fewer problem behaviours in their classrooms and will, therefore, use fewer coercive resources to address those problems (H2). Finally, the third hypothesis envisages that the relation and weight amongst these variables will be significantly different between primary and secondary education (H3).

## 2. Materials and Methods

### 2.1. Participants

This research paper forms part of the “Study on Coexistence in Aragón: situation, good practices and improvement proposals”. It forms part of the 1st Integral Plan against bullying at schools in the Spanish Autonomous Community of Aragón, Decree No. ECD/715/2016, in which 831 male/female teachers from 63 learning institutions took part and were randomly selected via sampling. Among them, 480 taught secondary education and 351 taught primary education. A percentage of 77.6% taught in public education, and 22.7% in privately run schools funded by the State. Among the teachers, 35.5% were male and 64.5% female. A relation was found between both variables (χ^2^ = 9.144, *p* = 0.002), and the percentage of female primary education teachers was somewhat higher (70.4%) than female secondary education teachers (60.2%).

Those who participated in this study had ample teaching experience: 56.4% had more than ten years teaching experience, 29.7% had been teachers for more than twenty years, 33% had been in the profession for two to nine years; only 10.6% were in their first teaching year. Apart from their professional experience, 97.6% had performed additional training activities in various areas of expertise, such as didactic specialisation, new technologies, conflict solving, coexistence, and so on.

### 2.2. Variables and Instruments

The variables herein included form part of a set of those that were proposed for a national study conducted in Spain in 2011 on the subject of coexistence at school [44]. All the scales in that study were validated by experts prior to application; they formed part of a design based on statistical analyses based on descriptive studies and exploratory factor analysis (EFA) [44]. Seven of the blocks therein employed were used as the starting point for the present study. Five of the seven blocks were considered possible exogenous variables (i.e., relationship amongst teachers; relationship between teachers and students; coexistence rules; teaching competences in motivational and inclusive teaching; and using participative and inclusive activities). The other two were employed as endogenous variables (i.e., student misbehaviour and teacher misbehaviour). All the items were designed on a Likert-type scale with four response options based on frequency: never, sometimes, often, and very frequently.

The set of items in our study was submitted to a new validation procedure, run by successively applying techniques based on exploratory factor and confirmatory analyses (ESEM), and applied to two subsamples randomly culled from all participants. This procedure is described in further detail in the statistical procedures section, prior to which the study variables are presented.

#### 2.2.1. Exogenous Variables

In relation to the ESEM performed with the predictor variables, the set of 37 items included in this block fits the six-factor model quite well insofar as four of the variables corresponded to the previously defined variables [44]. Meanwhile, the other one (“relationship amongst teachers”) was subdivided into two factors. Both the ESEM with six factors (χ^2^ = 1526.783, d.f. = 459; *p* < 0.001; root mean square error of approximation (RMSEA) = 0.053; comparative fit index (CFI) = 0.949; Tucker-Lewis index (TLI) = 0.926); and the confirmatory factor analysis (CFA) (χ^2^ = 1274.568, d.f. = 641; *p* < 0.001; RMSEA = 0.051; CFI = 0.934; TLI = 0.928) improved the results of the models with five factors: ESEM (χ^2^ = 1406.842, d.f. = 619; *p* < 0.001; RMSEA = 0.055; CFI = 0.932; TLI = 0.908) and CFA (χ^2^ = 1918,261, d.f. = 491; *p* < 0.001; RMSEA = 0.059; CFI = 0.921; TLI = 0.915).

The predictor variables herein employed are as follows:

*Relationship among teachers.* This block contains eight items, which, after the aforementioned analyses, comprised two variables: a first one referring to *Teachers’ perceived peer support* (F1) with four items (example: “I can count on help from other male/female teachers if I need it”). The second variable refers to *Teacher conflicts* (F6), represented by four items, including perceived teacher isolation and teachers’ poor peer relationships (example: “My colleagues don’t speak well of me”). Both ESEM and CFA confirm these two different factors: teachers’ perceived peer support (Rho = 0.900; average variance extracted (AVE) = 0.694) and teacher conflicts (Rho = 0.787, AVE = 0.485).

*Teacher–student collaboration* (F2). This indicator comprises five items that reflect an environment with good relationships and trust between teachers and students (example: “Students can count on their teachers to solve a conflict fairly”). After the ESEM and CFA results, the items corresponding to this indicator comprised four items that reflect an environment with good relationships between teachers and students, plus another one, “students stick to rules”, associated with coexistence rules, possessing a good fit (Rho = 0.883, AVE = 0.602).

*Coexistence rules*. Here, nine items appear in relation to perceiving that rules work (F3); that is, the teachers’ views regarding the fairness of coexistence rules, the students’ participation in the establishment of those rules, and the fulfilment of punishment (example: “Rules are fair”). Analysis results backed the composition of this factor (Rho = 0.880, AVE = 0.456).

*Teaching competence* (F4). This block includes five items whose contents are related to teachers’ capacity to provide motivational and inclusive teaching in a pleasant classroom environment (example: “I am able to make classes interesting”). Both ESEM and CFA upheld this factor’s independence (Rho = 0.857, AVE = 0.457), despite the appearance of certain inclusive teaching factors in the next factor.

*Use of participative and inclusive activities* (F5). This block comprises ten items associated with the use of various inclusive teaching resources in classrooms to encourage student participation in learning, peer collaboration, debate, consensus, cohesion, and inclusion (examples: “In class, I encourage my students to discuss social and political matters on which a range of views exist”; “I include activities based on student cooperation”). Both ESEM and CFA differentiate this factor, which is particularly associated with activities performed in classrooms (Rho = 0.906, AVE = 0.495), from the previous block, which refers more to teacher’s perceived personal competence.

#### 2.2.2. Endogenous Variables

For the criterion variables, the analyses revealed a good fit with two factors (χ^2^ = 403,699, d.f. = 134; *p* < 0.001; RMSEA = 0.049; CFI = 0.973; TLI = 0.966). The CFA run with the second subsample reproduced the same aforementioned factorial structure, likewise with acceptable fit indicators (χ^2^ = 527.843, d.f. = 151; *p* < 0.001; RMSEA = 0.078; CFI = 0.934; TLI = 0.925).

*Student misbehaviour with teachers* (F7). This includes ten items covering the aggressive behaviour towards teachers on the part of students who are less serious about learning; for example: “They are a nuisance and prevent me from giving the lesson” (Rho = 0.942; AVE = 0.629).

*Teacher coercion* (F8). This indicator comprises eight questions regarding problems with the way in which teachers deal with students, expressed as not paying attention, as well as rejection or coercion shown towards a male/female student in the past two months. Answering to these items, teachers had to indicate whether they had dealt with any student in the past two months by, for example, “not allowing him/her to participate in class” (Rho = 0.739; AVE = 0.309).

### 2.3. Procedure

Several schools located within the territory of the Autonomous Community of Aragon were randomly selected via quota convenience sampling. An invitation letter was sent to schools, containing information regarding the study’s main goals, and establishing the need for parental authorisation and informed consent. Research team members who were working in each city/town coordinated and supervised this phase by keeping in touch with all schools in person and via telephone. Once consent from a school was confirmed, it received usernames and passwords to access the online survey. The students from each school completed the survey under similar privacy conditions during school hours in a laboratory using computers. Anonymity and confidentiality were guaranteed. Each individual survey was completed in approximately twenty-five to forty minutes. This study was carried out in accordance with the recommendations of the Council of the British Educational Research Association in the second edition of their Ethical Guidelines for Educational Research [45]. Compliance with the standards of the Declaration of Helsinki on human experimentation was guaranteed at all times.

### 2.4. Data Analysis

Two aspects of the statistical procedure deserve particular interest: (1) the procedure we applied to validate the scales described in the Variables and Instruments section, and (2) the manner in which we established SEM models and intergroup comparisons. As indicated in the Variables and Instruments section, scales were validated by randomly dividing the whole sample of participants into two. The following were jointly considered in each of the two subsamples: those items related to the predictor variables, and those related to the criterion variable. Both were analysed, the first by ESEM and the second by CFA. We further maintained this mixed procedure, especially when a single alternative with ESEM could be valid [46], because we were working with a large sample that allowed the division into two independent validations and made them available for the application of the traditional validation procedure [46,47]. The estimation method that we followed referred to categorical variables, namely the weighted least square mean and variance adjusted (WLSMV) procedure of the above-cited package: the robust weighted least squares estimator. After establishing the resulting factorial structure, the validity indicators of the scales were obtained with the whole sample. Specifically, the following data are reported: construct validity (a recommended value of > 0.07): convergent validity by average variance extracted (AVE, a recommended value of > 0.05) [48,49]. In both cases, we employed the M-Plus statistical package to analyse data [50].

**Figure 1:**
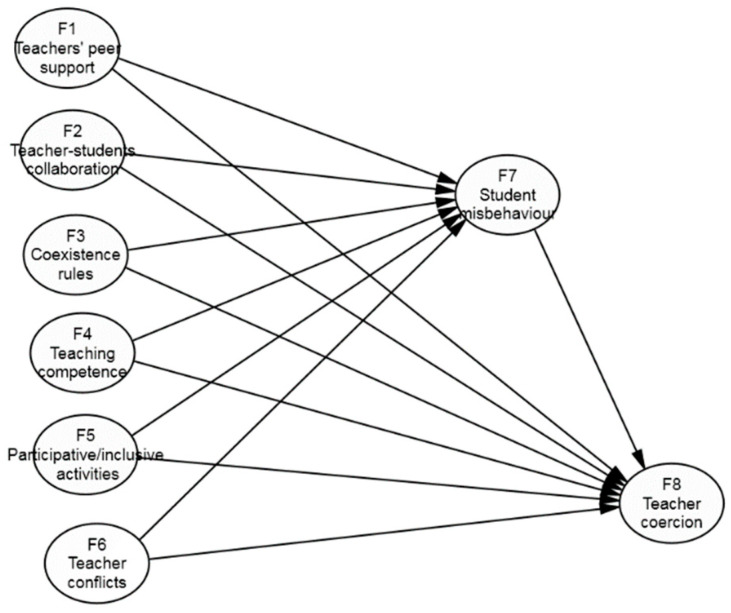
Theoretical model for SEM.

For the purpose of making intergroup comparisons (i.e., between two levels of education—primary versus secondary), we first obtained correlations among all the factor scores of the variables in both the Primary and Secondary Education teacher subsample. Then we made a comparison between them using Fisher’s Z transformation of the correlation coefficient. An SEM model was subsequently considered [47,51], which, along with the measurement models, added the possibility of establishing relations between variables by defining exogenous variables as the independent variables and endogenous variables as the dependent variables. In the same model, all of the exogenous variables were related to the endogenous variables, except for F1 (*teachers’ perceived peer support)* on F8 (*teacher coercion*), whose correlation was not significant in both the primary education and secondary education teacher subsamples. As pointed out in the theoretical framework, this model established that F7 (*student misbehaviour*) was an antecedent of F8 (*teacher coercion*). Thus, the endogenous variables could exert indirect effects on teacher coercion via student misbehaviour (Figure 1). Various models were compared with this model, distinct restrictions were determined for the equality of regression weights, and the measurement model of the latent variables remained stable.

Analyses were performed using M-Plus and the robust maximum likelihood (MLR) estimation method, which turns out to be the more appropriate method for making comparisons among groups in this programme. This estimation method is also robust to non-normality and non-independence of observations when applied to complex models such as this one. For the purpose of analysing the models’ fit, the fit indices reported by M-Plus were employed: the chi-square index and the normalised chi-square index (*χ*^2^/*DF*); RMSEA and SRMR (standardized root mean-square); TLI; and CFI [47,49,52]. When dealing with nested models, the comparisons of the models were carried out by calculating Δχ^2^ for the correction set by Satorra and Bentler [53] by means of Satorra-Bentler scaled chi-square (TRd) statistics and the Akaike information criterion (AIC) index [47].

## 3. Results

The correlations between factor scores are provided in Table 1. For F7 (*student misbehaviour with teachers*), the most frequently marked relations for both primary and secondary education were for well-perceived student–teacher relationships (F2) (primary, *r* = −0.561; secondary, *r* = −0.486) and also for teachers’ capacity to handle classes (F4), (primary, *r* = −0.471, secondary, *r* = −0.428). Both were associated with less disruptive student conduct. Certain major differences were found between both subsamples. The relationships amongst teachers evaluated on the basis of peer support (*r* = 0.020, ns.) and peer conflicts (*r* = 0.140, *p* = 0.035) were not relevant for primary education but were important for secondary education (*Z* = −4.24, *p* < 0.001; *Z* = 7.15, *p* < 0.001), while a lack of support (*r* = 0.272, *p* < 0.001) and conflicts (*r* = 0.348, *p* < 0.001) were related to more frequent disruptive conduct. In a similar vein, inclusive activities were related to student misbehaviour for secondary education (*r* = −0.247, *p* < 0.001) but not for primary education (*r* = −0.105, ns.). Finally, a small, albeit significant, correlation appeared in both cases, with no differences in subsamples found between student behaviour and the perception that the education centre’s norms were fulfilled. In addition, the education centre’s rules were more frequently perceived as not being met when more problematic conduct occurred (primary, *r* = 0.149; secondary, *r* = 0.243).

When analysing the correlation for teacher coercion (F8), a marked association with student misbehaviour appears, and it took place more often (*Z* = −4.83, *p* < 0.001) for primary education (*r* = 0.790 vs. *r* = 0.590). Once again, a close association between perceived good teacher–student collaborations (primary, *r* = −0.494; secondary, *r* = −0.212) and teaching competence (primary, *r* = −0.644; secondary, *r* = −0.257) can be noted. Teacher coercion was observed less frequently, albeit more often for primary education. The inclusive activities performed in classrooms were associated with both primary and secondary education, and with less teacher coercion (primary, *r* = −0.373, secondary, *r* = 0.248), the weight of which was heavier for primary than secondary education (*Z* = 1.97, *p* = 0.048). The education centre’s rules being perceived to work were related only to teacher coercion for primary education (*r* = −0.171). Peer support and peer conflicts also showed different profiles for primary and secondary education. Indeed, peer support was not significant in any case (Table 1), whereas peer conflicts were associated with less teacher coercion for primary education (*r* = −0.461), and slightly more for secondary education (*r* = 0.171).

The SEM model was tested by including the causal structure highlighted in our research objectives (Figure 1). It was also tested by comparing the different models, which analysed the possible differential role of the predictor variables on both levels of education. With the first model theoretically defined in Figure 1, two models were tested: Model 1 and Model 2. The first was used to consider the equality of all of the factor weights, whereas the non-equality in all of them was considered with the second. Between both models (Table 2), a better fit was found by considering differences in all of the regression weights (TRd = 27.8166, ∆*df*. = 12, *p* < 0.001). Equality restrictions for the various regression weights were imposed on this model by beginning with the Model 2 estimations. The models (Models 2.1, 2.2, and 2.3) that successively incorporated the equality of the regression weights (F7 on F8; F2 and F3 on F7) were checked, and this did not make the fit of model 2 any worse (*TRd* = 11.348, ∆*d.f.* = 3, *p* = 0.142, ∆ACI = −2.373). However, the fit of another model (Model 2.4), which added the equality of F4 on F7, became worse (*TRd* = 12.1065, ∆*d.f.* = 4, *p* = 0.067, ∆AIC = 4.195).

**Figure 2:**
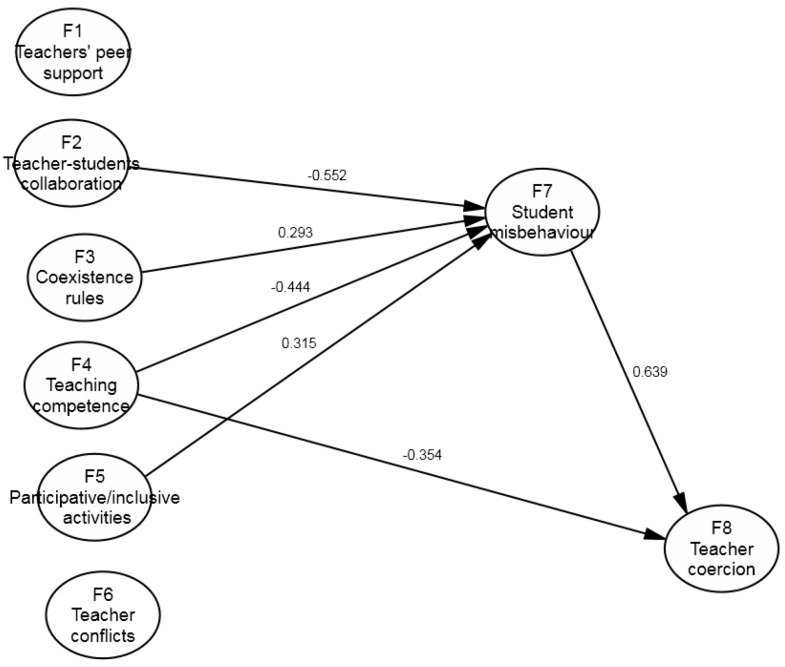
Results for primary school teachers.

**Figure 3:**
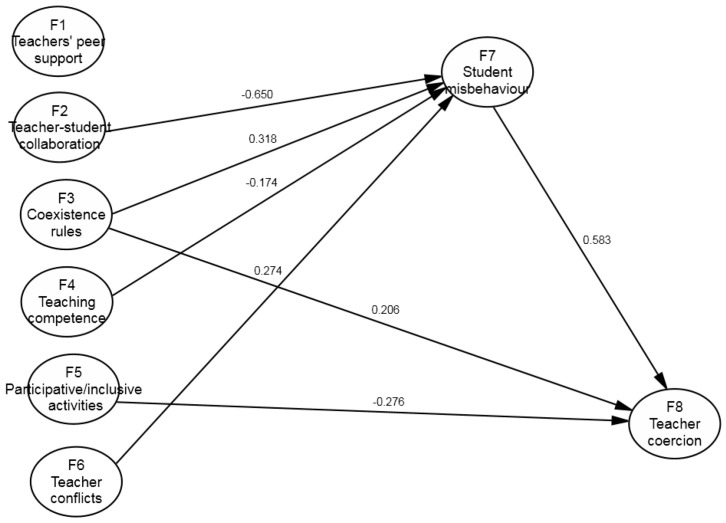
Results for secondary school teachers.

Following these comparisons, which affected the statistically significant regression weights in the two groups, the equality of the other regression weights was tested for those cases in which the Model 2 estimation of one of the subsamples was not statistically significant. In the series of Models 3.1 to 3.3, the value was restricted to 0 in both subsamples, whereas the equality of the regression weights was restricted with no previous value in the series of models 4.1 to 4.3. As seen in Table 2, all of these options made the model’s fit worse, so we were unable to make these restrictions and kept Model 2.3 as the one with the best fit. In Model 2.3, the values of the absolute fit measures, such as RMSEA (0.053), were adequate, as were other parsimonious fit measures, such as the normed chi-square (2.166). Meanwhile, the incremental fit values, such as TLI (0.744) and CFI (0.749), moved slightly away from the recommended values. Nonetheless, the incidence of these indices in the comparison of models led us to maintain this model, even though alternative models could be considered (as mentioned in the Discussion section). Figure 2 and Figure 3 show the results for the primary and secondary education groups. These models explain a similar percentage of variance for F7 (*student misbehaviour*) for primary (*R*^2^ = 0.360) and secondary (*R*^2^ = 0.378) education but not for F8 (*teacher coercion*), a variable that better explains such conduct for primary education (*R*^2^ = 0.759) than for secondary education (*R*^2^ = 0.465).

This model considers that teacher coercion (F8) is determined as mainly based on student misbehaviour with teachers, and this weight is not different in the two subsamples (*β* secondary = 0.583, *p* < 0.001); *β* primary = 0.639, *p* < 0.001). Certain differences appear in the other predictors: for instance, the large weight observed for primary education teacher competence (*β* = −0.354, *p* < 0.001) was not relevant for secondary education (*β* = 0.010, *p* = 0.937). For secondary education, however, we found that two other predictors influenced teacher coercion with students: performing inclusive activities in classrooms (*β* = −0.276, *p* = 0.007), and perceiving that rules worked (*β* = 0.207, *p* = 0.034), both of which were not significant variables in the primary education group.

Regarding perceived student misbehaviour with teachers (F7), a strong predictor of the same variables was found with the same weight in both subsamples, namely perceived good teacher–student relationships (*β* secondary, 0.650, *p* < 0.001); *β* primary, 0.552, *p* < 0.001). After this variable was found, and under equal conditions, the predictor “perceiving the centre’s rules being adhered to” stood out (*β* secondary, 0.318, *p* = 0.004; *β* primary, 0.293, *p* < 0.001). The other predictors differed between both subsamples. For secondary education, the higher frequency of problematic relationships with other teachers predicted student misbehaviour (*β* = 0.274, *p* = 0.001), and teaching competence was perceived less (*β* = −0.174, *p* = 0.037). For primary education, more inclusive activities were performed in classrooms (*β* = 0.315, *p* = 0.001), and the model’s predictive power increased.

The analysis of indirect effects allowed for a better understanding of the effects of the exogenous variables on the endogenous ones, specifically when it came to analysing which variables had effects on teacher coercion (F8) through F7 (*student misbehaviour*). In the secondary education teacher group, teacher–student collaboration (F2), competence with motivational and inclusive teaching (F4), and teacher conflicts (F6) have indirect effects on teacher coercion (*β* = −0.379, *β* = −0.102, *β* = 0.160). Coexistence rules (F3) became the best predictor among the exogenous variables (*β* = 0.392) by accumulating the direct (*β* = 0.207) and indirect (*β* = 0.185) effects. We found a similar situation in the primary education group, but with distinct variables. Three exogenous variables had indirect effects exclusively on teacher coercion (F8), teacher–student collaboration (F2, *β* = −0.352), coexistence rules (F3, *β* = 0.187), and the use of participative/inclusive activities (F5, *β* = 0.201). The best joint predictor for F8 was F4 (*competence with motivational and inclusive teaching*), which accumulated a total weight of *β* = −0.638. It also accumulated a direct effect of *β* = −0.354 and an indirect effect of *β* = −0.284.

## 4. Discussion

This study’s objective was to analyse student and teacher misbehaviour from the teacher perspective with a sample of Spanish teachers, in an attempt to compare the predictive differential value of certain contextual variables at the two most important levels of compulsory education in Spain: primary and secondary education. The relevance of the present study lies in the scarcity of such studies on an international level, especially in non-Anglo-Saxon countries. We now present and discuss this study’s most relevant results. We hypothesised that student misbehaviour would be the main predictor of teacher coercion (H1). We also hypothesised that teachers who maintained better relationships with their students would establish motivational and inclusive teaching strategies, would use a greater amount of didactic resources to encourage student participation, would have a sound perception of the coexistence rules, and would have a greater amount of support from peers. Thus, they would observe less problematic conduct during their classes and would resort to fewer coercive resources to address these problems (H2). The chosen sample type allowed us to compare primary and secondary education teachers, which allowed us, in turn, to verify our third hypothesis (H3).

We found a marked relation between student misbehaviour and teacher coercion, and we also found that student misbehaviour was an antecedent of teacher misbehaviour. The SEM model showed a causal relationship between these two variables. These results support H1. We were able to ascertain that the main source for explaining teacher coercion was the teachers’ reactions to student behaviour. Many sources have ascertained that teachers react to student behaviour, and such reactions constitute one of their main effects. Teachers’ attempts to deal with and to control student misbehaviour in classrooms can lead to inappropriate coercive reactions and responses on their part.

Regarding H2, our study supports the notion that teachers who perceive teacher–student collaborations feel competent as teachers and have the resources they require to perform inclusive and collaborative activities. They need to be able to count on such activities in order to be able to deal more suitably with challenges associated with the demands arising from student misbehaviour. This helps with collaborating with peers, especially in the secondary education setting, whereas trust in rules does not generally have much weight for predicting teachers’ actions. Teacher coercion is stressed as being unsuitable and is, to a great extent, a response to student misbehaviour that should be replaced with other alternatives. In short, our Hypothesis 2 would be confirmed for the most part, both in primary and secondary education. This study did not focus on analysing the frequency of coercive conduct. However, in line with Clunies-Ross et al. [29], we believe that coercive behaviour is not the most frequent type of conduct in the Spanish context. What is relevant is the continued lack of knowledge as well as of teacher competence, which prevents alternative strategies from being adopted [9,17,29,35]. Thus, it is essential to insist on the need for improved teacher training, even though developing teacher competence is difficult in this context because either initial training curricula lack content [7] or because it is difficult to implement such training in practice [7,54,55]. One alternative would be to gain a better grasp of the social context in which this competence is developed in order to be able to address these situations, which involves not only teacher training but also the context of the education centre [32]. This is what Klassen [37] refers to when talking about building a collective sense for dealing with these situations.

With regard to H3, the analysis performed on the correlations revealed a slight difference insofar as the relation between student misbehaviour and teacher coercion was found to a greater extent for primary education. However, as the SEM model did not validate such differences, we can state that this relation was well established at both levels of education. Roache and Lewis [11] also found that primary and secondary education teacher groups adopted an equal coercion style when faced with problematic student behaviour, which supports the idea of equality between both groups. However, the abovementioned authors found differences in other variables that we did not include herein—for example, the types of proactive strategies that teachers adopt, and teachers’ concerns stemming from various types of student conduct. Beaman et al. [27] also mentioned the latter finding. Kulinna [15] reported differences between primary and secondary education teachers when they analysed the attributive style of student misbehaviour. She tended to make more attributions to external school factors for secondary education students than for primary education students. As we did not include this variable in our study, we were unable to compare the results. However, we believe that these attributions could lead to more differences in proactive-type strategies than in the coercive ones analysed in the present study. The study by Bear et al. [20] followed this same line, as those authors found a greater use of rewards for primary education students than for secondary education ones, and those practices had a stronger positive/negative impact on secondary education students.

Regarding the relevance of the contextual variables, most of the variables included in our study support their relevance as preventive measures against student misbehaviour as indicated in the theoretical introduction at the beginning of this paper. Nevertheless, we found some differences between primary and secondary education, which support our H3 hypothesis. On the one hand, on both levels of education, the SEM model highlighted the importance of teacher–student collaboration as an element inversely associated with student misbehaviour. On the other hand, two dimensions related to how teachers act are also relevant: the use of participative and inclusive activities, as well as teaching competence. We observed that the relevance of perceived classroom management was greater for primary education than for secondary education. This reinforces the idea that perceived teaching competence would be more important for preventing problematic conduct in primary education classrooms, which is a novel outcome.

These variables also had differential weights in the course of the analysis of teacher coercion. We specifically found that perceived classroom management predicted less frequent teacher misbehaviour in both cases, and it supported the authors’ data by indicating that perceived teacher competence and the types of strategies teachers adopt tend to mediate the reactive strategies they adopt in order to address student behaviour [32]. In our study, the only significant predictor for primary education was “perceived teacher competence in classrooms”, whereas the “type of inclusive activities” variable was added for secondary education.

These results, in addition to our H3 hypothesis, also support the hypothesis proposed by Clunies-Ross et al. [29], namely that the educational level at which teachers teach could be an important variable for understanding the relations between the role of teachers’ peer relationships and their types of reactions when faced with problematic student conduct. More specifically, teacher–student collaborations—that is, good relationships—did not show any differences between primary and secondary education in the SEM model. However, the other variables did, such as teaching competence or the activities performed in the classroom.

Regarding the value of rules in accordance with teachers’ perceptions, we found that these elements were directly related to the frequency of student problematic conduct in the case of both primary and secondary education. This relation should indicate the kinds of rules that prevail in education centres from the teachers’ perspectives, as well as the feeling of being dissatisfied with these rules when conduct problems more frequently arise. No former studies had made these comparisons between primary and secondary education.

Finally, another difference appeared for student misbehaviour when the models were compared: conflictive peer relationships were associated with more frequent student misbehaviour in secondary education. The correlations even indicated a positive weight for primary education, but this variable was not significant in the SEM model. Thus, peer relationships in the primary education setting could be associated with either teaching competence or the kinds of activities performed in classrooms, since the normal practice is to undertake educational projects that affect a group of teachers in the same course or the same subject. Let us not forget that teacher relationships might also depend on the education centre’s culture, as Clunies-Ross et al. [29] have likewise pointed out. Nevertheless, these data cannot be compared with those reported elsewhere. Tsouloupas et al. [32] did indeed contemplate the relevance of support from peers and school administrative teams for American teachers’ self-efficacy in handling student misbehaviour. Still, these authors neither made comparisons nor hypothesised this. Our data, thus, needs to be replicated in other studies.

Our study has certain limitations. The first one is related to the fact that the measures we included come from self-report sources and may contain biases. However, observational studies that have analysed the relation between self-reported and observational measures [3,29] uphold the value of self-reports. Given the cross-sectional nature of this study, and even though SEM models allow causal relations to be established at least statistically, its design would not allow this causal relation to be considered in the strictest sense. In addition, some opposite relations could even appear for instance: a greater amount of students could misbehave; thereby conditioning the types of classroom practices being implemented, teaching competence, and the assessment of rules. In other cases, such as analysing teaching self-efficacy to handle student misbehaviour, the same kind of problem has been explored [7]. We were mainly interested in establishing relations between student misbehaviour and teacher coercion, as well as elucidating how certain contextual variables can directly or indirectly affect these relations and teachers’ responses when they address student misbehaviour. Thus, we considered these relations in a single direction, in order to determine the explanatory power of the different predictor variables explored in our study.

Finally, in future analyses, we shall consider the relevance of designing longitudinal studies that could consider changes in student conduct and teachers’ responses to student misbehaviour more accurately as a result of changes in the relations amongst certain variables contemplated herein. It would likewise be of great interest for us to explore these same relations from a student perspective.

## 5. Conclusions

To improve harmonious coexistence in educational institutions, the relations between the teaching staff and the students need to be taken into account. In this study, we analysed manifestations of conflictual behaviour displayed by teachers as well as students in their daily interactions—e.g., student misbehaviour and teacher coercion—according to the self-report data provided by teachers in centres of primary and secondary education. Our results show that the two variables, student misbehaviour and teacher coercion, are strongly related, but that further variables help to prevent or, conversely, contribute to enhance the probability of such conduct. Particularly, when teachers have a favourable perception of their own competence, it helps them establish collaborative relations with the students, thereby leading to mutual support among colleagues, strategies of cooperation and participation among students, and a lower incidence of conflictual behaviour.

On the other hand, this study has practical implications. It is well known that one of the greatest sources of stress for teachers is the fact that they have to deal with student behaviour in the classroom [3,11,12]. Teachers often try to confront such situations with inadequate strategies, which leads them to adopt coercive behaviour themselves, thereby engendering a vicious circle. This study, thus, has a series of practical implications that can lead us to reflect on our own teaching practices, while also inspiring us to improve them by taking the different dimensions of school climate analysed herein into account. Teachers need to possess a series of skills and resources that allow them to establish good relations with students as well as with their colleagues; moreover, they need to develop a series of good teaching practices that help them reinforce their feeling of being competent, thereby allowing them to attend to the multiple needs of different types of students present in the classroom. Furthermore, it is important to analyse the context in which the teacher carries out his/her activity: the present study revealed significant differences between primary and secondary education teachers in relation with classroom context and, more generally, in relation with teacher peer support, and in relation with each educational centre’s system of norms and rules. The training of emotional competences [42] and of specific competences [28] has been shown to be a good alternative, but that task should not be left in the hands of the teachers alone. It also needs to involve the centres’ administrative teams as well as government organisms, along with all professors/educators who are in charge of training teachers on an initial and/or ongoing level.

## Figures and Tables

**Table 1:** Factor score correlations.

Primary School Teachers, n = 351							
	F1	F2	F3	F4	F5	F6	F7
F1	1						
F2	0.463 ***						
F3	0.598 ***	0.681 ***					
F4	0.194 **	0.492 ***	0.308 ***				
F5	0.180 **	0.325 ***	0.248 ***	0.640 ***			
F6	−0.627 ***	−0.148	−0.437 ***	0.131	0.107		
F7	0.020	−0.561 ***	−0.149 *	−0.471 ***	−0.105	−0.140 *	
F8	−0.032	−0.494 ***	−0.171 **	−0.644 ***	−0.373 *	−0.461 ***	0.769 ***
**High School Teachers, n = 480**							
F1	1						
F2	0.524 ***						
F3	0.548 ***	0.678 ***					
F4	0.339 ***	0.474 ***	0.342 ***				
F5	0.275 ***	0.370 **	0.347 ***	0.658 ***			
F6	−0.531 ***	−0.211 ***	−0.387 ***	−0.189 **	0.104		
F7	−0.272 ***	−0.486 ***	−0.243 ***	−0.428 ***	−0.247 ***	0.348 *	
F8	−0.106	−0.212 **	−0.025	−0.257 ***	−0.248 ***	0.171 ***	0.590 ***

Note: F1 = Teachers’ perceived peer support; F2 = Teacher–student support; F3 = Coexistence rules; F4 = Teaching competence; F5 = Use of participative and inclusive activities; F6 = Teacher conflicts; F7 = Student misbehavior (with teachers); F8 = Teacher coercion. *** *p* < 0.001; ** *p* < 0.01; * *p* < 0.05.

**Table 2:** Fit indexes for structural equation models.

	Model	CMIN	DF	*p*	CMIN/DF	CFI	TLI	RMSEA	SRMR	AIC
1	MODEL 1	6577.912	3030	0.000	2.171	0.747	0.743	0.053	0.085	6,3561.851
2	MODEL2	6540.926	3018	0.000	2.167	0.749	0.743	0.053	0.083	6,3542.653
3	MODEL 3	6587.720	3030	0.000	2.174	0.746	0.742	0.053	0.085	6,3570.009
4	MODEL 4	6590.956	3033	0.000	2.173	0.746	0.742	0.053	0.086	6,3572.299
5	MODEL 2.1	6541.109	3019	0.000	2.167	0.749	0.744	0.053	0.083	6,3541.394
6	MODEL 2.2	6542.986	3020	0.000	2.167	0.749	0.744	0.053	0.083	6,3542.234
7	MODEL 2.3	6542.182	3021	0.000	2.166	0.749	0.744	0.053	0.083	6,3540.280
8	MODEL 2.4	6550.091	3022	0.000	2.167	0.748	0.743	0.053	0.084	6,3546.848
9	MODEL 3.1	6563.312	3024	0.000	2.170	0.748	0.743	0.053	0.084	6,3555.630
10	MODEL 3.2	6560.399	3024	0.000	2.170	0.748	0.743	0.053	0.084	6,3555.635
11	MODEL 3.3	6558.053	3024	0.000	2.169	0.748	0.743	0.053	0.084	6,3551.102
12	MODEL 3.4	6563.711	3024	0.000	2.171	0.747	0.743	0.053	0.084	6,3558.635
13	MODEL 4.1	6550.299	3023	0.000	2.167	0.748	0.744	0.053	0.084	6,3545.860
14	MODEL 4.2	6557.561	3023	0.000	2.169	0.748	0.744	0.053	0.084	6,3553.504
15	MODEL 4.3	6550.867	3023	0.000	2.167	0.748	0.744	0.053	0.084	6,3545.821

Note: Model 1: All weights are equal when one eliminates the variables that do not statistically differ from zero in the bivariable correlations (F1 on F8); Model 2: The same as Model 1, but leaving all the estimations between primary and secondary teachers free; Model 3: The SEM model built on Model 2 by setting the weights that do no statistically differ from zero in the estimation to zero; Model 4: The SEM model built by making all the regression weights equal that were not zero in Model 3; Model 2.1: All weights are free, except for F7 on F8, which is equalled. The other variables on F8 differ; Model 2.2: we add another restriction to Model 2.1, this time with the equality of F2 on F7; Model 2.3: We add another restriction to the previous model, making F3 on F7, F7 on F8, F2 on F7 and F3 on F7 equal; Model 2.4: We add another restriction to the previous model with F4 on F7; Model 3.1: By taking Model 2.3 as a reference, we add restrictions with weights that equal zero, specifically making F8 on F5 equal zero in both groups; Model 3.2: The same as Model 3.1, but with F4 on F8 instead of F8 on F5; Model 3.3: The same as Model 3.1, but with F5 on F7 (equalling zero); Model 3.4: The same as Model 3.1, but with F6 on F7 (equalling zero); Model 4.1: As in Model 3.1, but with a set weight that is not necessarily 0; Model 4.2: As in Model 3.2, but with a set weight that is not necessarily 0; F8 on F4; Model 4.3: As in Model 3.3, with F5 on F7 being equal but not equal to zero. CMIN = related Chi-square statistics; DF = degrees of freedom; CFI = comparative fit index; TLI = Tucker-Lewis index; RMSEA = root mean square error of approximation; SRMR = standardized root mean-square; AIC = Akaike information criterion.
